# *Streptococcus intermedius* lung infection masquerading as malignancy

**DOI:** 10.1002/ccr3.8018

**Published:** 2023-10-09

**Authors:** Saliha Saleem, Taylor B. Nelson

**Affiliations:** ^1^ University of Missouri Division of Infectious Diseases Columbia Missouri USA

**Keywords:** lung abscess, lung mass, malignancy, *Streptococcus intermedius*

## Abstract

*Streptococcus intermedius* can cause aggressive infections. One such example as reported in this case is lung abscess which may be mistaken as malignancy on lung imaging.

## Graphical abstract

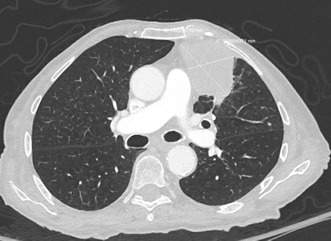

A 77‐year‐old female with Parkinson's disease and chronic obstructive pulmonary disease presented to the hospital due to worsening exertional shortness of breath and productive cough for several days. She had a 50 pack‐year history of smoking cigarettes. On presentation, she was hemodynamically stable and required four liters of oxygen, which was new for her. Initial laboratories included complete blood count and comprehensive metabolic profile which were within normal limits. Computerized tomography (CT) of chest with intravenous contrast showed a 3.0 cm × 2.7 cm mass within the lingula and a larger second mass with poorly defined margins in left upper lobe measuring 6.7 cm × 4.8 cm × 6.3 cm. This mass abuted the mediastinal margin, the anterior mediastinal fat, and anterior chest wall. Ipsilateral left mediastinal lymphadenopathy was noted (Figure 1). Malignancy was suspected given her age, smoking history, and appearance of the masses on imaging. A CT‐guided biopsy and bronchoscopy with bronchoalveolar lavage (BAL) were performed. Cytology from both lung biopsy and BAL were negative for malignant cells. Fungal culture and acid‐fast bacillus (AFB) culture were negative from BAL. Culture from the BAL grew *Streptococcus intermedius (S. Intermedius)*. She was discharged on 4 weeks of amoxicillin/clavulanic acid and four liters of continuous oxygen. At follow‐up, she was clinically improved, with no oxygen requirements. Her repeat CT chest done after 4 weeks which showed significant reduction in size of the masses. She was continued on amoxicillin/clavulanic acid for another 4 weeks, after which repeat lung imaging showed near resolution of lingular mass and further reduction in size of left upper lobe mass. Antibiotics were discontinued after 8 weeks of total treatment.

**FIGURE 1:**
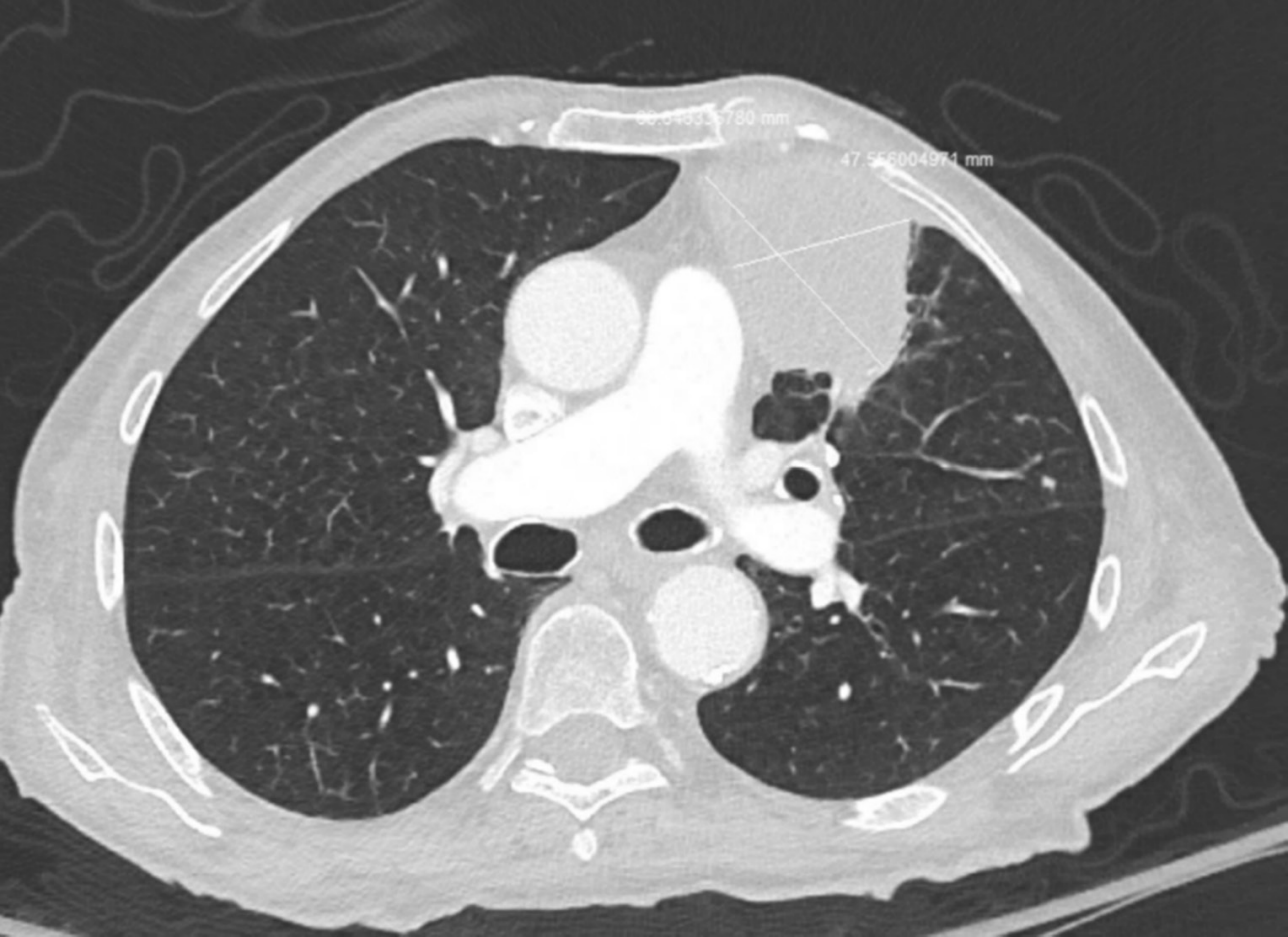
CT scan showing lingular mass abutting the mediastinal margin, mediastinal fat, and chest.

*Streptococcus anginosus* group (SAG) consists of three different species: *S. Anginosus*, *S. Intermedius*, and *S. Constellatus*. SAG species have been considered opportunistic pathogens to some extent, and risk factors include but are not limited to periodontal disease, malignancy, type two diabetes mellitus, chronic kidney disease, chronic lung disease, tobacco smoking, liver disease, and central nervous system disease. *S. intermedius* has been reported in brain abscesses, orofacial abscesses, pleural empyema, pneumonia, pericarditis, endocarditis, and liver abscess.^1^ *S. intermedius* is particularly prone to causing abscesses and deep tissue infections.^1^, ^2^, ^3^ This case describes a lung mass in an elderly female which eventually cavitated to form an abscess. Given the strong smoking history, advanced age, and imaging findings, malignancy was suspected, but cultures grew *S. intermedius* and cytology was negative. Her repeat imaging showed remarkable improvement with antibiotics. *S. intermedius* can cause invasive pulmonary infections mimicking malignancy and should always be considered in the differential for a lung mass. Excellent clinical recovery has been reported with appropriate antibiotics. To summarize this case presents an elderly female with significant smoking history and lung mass. Lung mass as per CT imaging appeared invading the surrounding tissue and mediastinum, which led to high concern for malignancy. The biopsy, however, ruled out malignancy and showed *S. Intermedius*. Repeat imaging revealed resolution of mass with antibiotics.

## AUTHOR CONTRIBUTIONS

**Saliha Saleem:** Writing – original draft; writing – review and editing. **Taylor Nelson:** Supervision; writing – review and editing.

## FUNDING INFORMATION

No funding was obtained for this article.

## CONFLICT OF INTEREST STATEMENT

The authors declare that there is no commercial or financial relationship to this article.

## CONSENT

Patient written consent was taken for the publication of images and patient related information. No patient identifiers were used.

## ETHICS STATEMENT

No ethical approval was required for this article.

## Data Availability

The data that support the findings of this study are openly available in the following references.^1^, ^2^, ^3^
